# Identification of hair shaft progenitors that create a niche for hair pigmentation

**DOI:** 10.1101/gad.298703.117

**Published:** 2017-04-15

**Authors:** Chung-Ping Liao, Reid C. Booker, Sean J. Morrison, Lu Q. Le

**Affiliations:** 1 Department of Dermatology,; 2 Department of Pediatrics,; 3 Children's Research Institute,; 4 Simmons Comprehensive Cancer Center,; 5 Hamon Center for Regenerative Science and Medicine,; 6 Howard Hughes Medical Institute, University of Texas Southwestern Medical Center, Dallas, Texas 75390, USA

**Keywords:** stem cell factor (SCF), hair pigmentation, hair shaft progenitor cell, hair follicle stem cell, hair matrix, KROX20

## Abstract

Hair differentiates from follicle stem cells through progenitor cells in the matrix. In contrast to stem cells in the bulge, the identities of the progenitors and the mechanisms by which they regulate hair shaft components are poorly understood. Hair is also pigmented by melanocytes in the follicle. However, the niche that regulates follicular melanocytes is not well characterized. Here, we report the identification of hair shaft progenitors in the matrix that are differentiated from follicular epithelial cells expressing transcription factor KROX20. Depletion of *Krox20* lineage cells results in arrest of hair growth, confirming the critical role of KROX20^+^ cells as antecedents of structural cells found in hair. Expression of stem cell factor (SCF) by these cells is necessary for the maintenance of differentiated melanocytes and for hair pigmentation. Our findings reveal the identities of hair matrix progenitors that regulate hair growth and pigmentation, partly by creating an SCF-dependent niche for follicular melanocytes.

## Abstract (precis)

Liao et al. report the identification of hair shaft progenitors in the matrix that are differentiated from follicular epithelial cells expressing transcription factor KROX20. Expression of stem cell factor (SCF) by these cells is necessary for the maintenance of differentiated melanocytes and for hair pigmentation.

Hair is a highly keratinized tissue differentiated from hair follicle (HF) stem cells, which are located primarily in a discrete reservoir called the bulge (Cotsarelis et al. 1990; Blanpain et al. 2004; Tumbar et al. 2004). The cycle of hair growth and involution occurs in three phases: anagen (growth), catagen (regression), and telogen (resting) (Blanpain and Fuchs 2009; Shimomura and Christiano 2010; Plikus et al. 2012; Solanas and Benitah 2013; Watt 2014). Hair is commonly pigmented because of the presence of melanin in hair shafts, which in turn is synthesized by follicular melanocytes and then transferred to the neighboring hair progenitors that give rise to hair shafts (Nishimura et al. 2002, 2005; Lin and Fisher 2007). Despite recent advances in our understanding of HF stem cell biology, the mechanisms that regulate hair pigmentation remain poorly understood, especially with respect to the cellular interactions between hair progenitor cells and melanocytes that take place in the HF matrix.

Stem cell factor (SCF, also known as KIT ligand or Steel factor) is a growth factor that regulates multiple physiological homeostatic events, including the maintenance of hematopoietic stem cells (Ding et al. 2012), mast cells (Lennartsson and Ronnstrand 2012), and melanocytes (Lennartsson and Ronnstrand 2012). The critical role of SCF and its receptor, KIT, for hair pigmentation has been supported by the absence of hair pigment in several SCF- and KIT-deprived animals (Copeland et al. 1990; Reith et al. 1990; Kunisada et al. 1998b; Botchkareva et al. 2001; Nishimura et al. 2002). It is apparent that melanocytes are the relevant target cells in this type of hypopigmentation, as they are the melanin-producing cells as well as the predominant KIT-expressing cell in the HF. However, the sources of SCF that support melanocytic activity in the HF have not been fully characterized, although SCF expression by cells in the dermal papilla has been suggested to promote melanocyte stem cell differentiation (Chang et al. 2013).

The HF matrix is composed of epithelial cells surrounding the dermal papilla. The HF matrix cells proliferate and differentiate into the structures of the hair shaft and inner root sheath (Driskell et al. 2011). There are also melanocytes residing in the same niche to provide melanin to hair shaft progenitor cells for hair pigmentation. A dominant population of matrix cells is transit-amplifying cells. They proliferate rapidly for several divisions and then progress upward to become committed progenitors that differentiate into central hair shafts (the medulla, cortex, and cuticle), its surrounding channels or the inner root sheaths (the cuticle, Henle, and Huxley layers) that guide hair shafts toward the skin surface, and the companion layer (the innermost layer of the outer root sheath) (Hsu et al. 2011, 2014). In contrast to HF stem cells in the bulge, the identities of the committed progenitor cells that give rise directly to hair shafts (the medulla, cortex and cuticle) are currently not well characterized. In addition, the development of hair is also accompanied by pigmentation that is contributed to the hair shafts by melanocytes. The reciprocal interactions between melanocytes and hair progenitor cells as well as the source of SCF, which regulates hair pigmentation, are not well characterized.

KROX20 (also known as EGR2) is a zinc finger transcription factor that regulates hindbrain segmentation (Schneider-Maunoury et al. 1993), Schwann cell myelination (Topilko et al. 1994), and lymphocyte immune responses (Li et al. 2012). In addition, KROX20 is expressed in subpopulations of HF cells (Gambardella et al. 2000). However, the role of the KROX20-expressing cells in HF development is not known. We now report that the transcription factor KROX20 identifies a sublineage of HF epithelial cells toward the differentiation of hair shaft during HF morphogenesis. We observed that they are a necessary source of SCF for the maintenance of mature melanocytes in the hair matrix to produce hair pigmentation, as evidenced by the complete loss of pigment when *Scf* is deleted in *Krox20* lineage cells. We also demonstrate that deletion of *Krox20* lineage cells in vivo results in a complete arrest of new hair growth, demonstrating the critical role of *Krox20* lineage cells in hair development.

## Results

### Mice lacking SCF in *Krox20* lineage cells exhibit progressive hair graying

**Figure 1.**
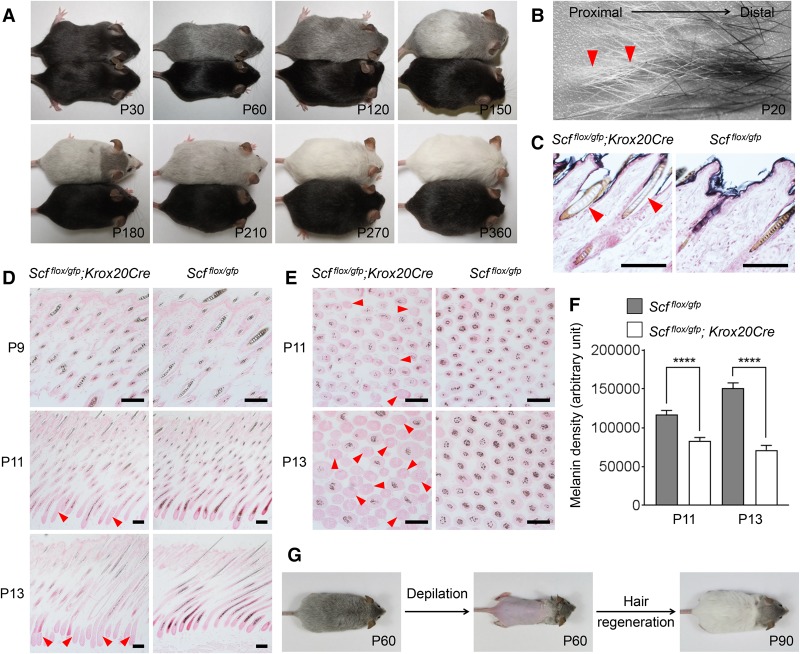
Mice lacking SCF in *Krox20* lineage cells exhibit progressive hair graying. (*A*) A representative *Scf^flox/gfp^; Krox20Cre* mouse (*top*) displaying hair graying along with aging as compared with a littermate *Scf*^*flox/gfp*^ control (*bottom*). *n* = 20. (*B*) Pelage hairs plucked from P20 *Scf^flox/gfp^; Krox20Cre* mice showed distinct pigment levels in distal and proximal ends; newly developed hairs in the proximal side (arrowheads) were absent of pigment. (*C*) Fontana-Masson staining confirmed the absence of melanin in the newly synthesized hairs (arrowheads) in P32 *Scf^flox/gfp^; Krox20Cre* mice. (*D*) HF section revealing normal hair pigmentation in *Scf^flox/gfp^; Krox20Cre* mice at P9 followed by a rapid loss of pigmentation beginning from the hair matrix (arrowheads) at P11 and P13. (*E*) Transverse HF section revealing hypopigmented hair shafts (arrowheads) in *Scf^flox/gfp^; Krox20Cre* mice at P11 and P13. (*F*) Quantification and statistical analysis of *E* showing a significant reduction of melanin density in *Scf^flox/gfp^; Krox20Cre* graying hair shafts. Melanin density was analyzed by ImageJ. Data are mean ± SEM from 100 randomly selected HFs in each group. (****) *P* < 0.0001. (*G*) Gray *Scf^flox/gfp^; Krox20Cre* mice were depilated to stimulate hair regeneration; new hair shafts were completely absent of pigment. *n* = 3. Nuclei were stained with nuclear Fast Red in *C*–*E*. Bar, 100 µm.

While studying the role of mast cells and SCF during the initiation of neurofibroma, a Schwann cell neoplasm, we conditionally deleted *Scf* in neurofibroma neoplastic cells using the Schwann cell lineage *Krox20Cre* mouse line. Serendipitously, we found that the mice without SCF in *Krox20* lineage cells developed hair graying and, early in life, lost all hair pigmentation. Invariably, all *Scf^flox/gfp^; Krox20Cre* mice (*n* = 20) displayed progressive hair graying. The first round of hair graying occurred homogenously during postnatal days 30–40 (P30–P40). As the mice aged, the hairs underwent further depigmentation in waves. This change converted all black pigmented hairs to white within 9 mo (Fig. 1A; Supplemental Fig. S1A). We believe that this pattern of hair color change is associated with the mouse hair cycle when old hairs are replaced by newly generated hairs (Plikus and Chuong 2008; Shimomura and Christiano 2010).

To characterize the cause of hair graying in the *Scf^flox/gfp^; Krox20Cre* mice, we first analyzed the pelage hair shafts plucked from P20 dorsal skin. Interestingly, the amount of pigment at the distal and proximal ends of each hair shaft was quite different; the amount of pigment at the distal appeared normal, but the pigment was mostly absent from the proximal end (Fig. 1B). Fontana-Masson staining confirmed that this hypopigmentation was caused by the absence of melanin in the hair shaft cortex and medulla (the compartments housing the most melanin in pigmented hair shaft) (Fig. 1C). We then determined the time of onset of decreased pigmentation. The amount of pigment in individual hairs appeared normal at P9 (Fig. 1D), with the loss of hair pigmentation becoming noticeable at P11; it progressed quickly after that (Fig. 1D,E). Analysis of melanin density in the HF revealed significant reductions at P11 and P13 (*P* < 0.0001) (Fig. 1F), explaining the early loss of hair pigmentation in *Scf^flox/gfp^; Krox20Cre* mice (Fig. 1B). *Scf^flox/gfp^; Krox20Cre* mice exhibited progressive hair graying; their coat color underwent a dynamic change from black to white within 9 mo (Fig. 1A; Supplemental Fig. S1A). However, their newly synthesized hairs were already largely depigmented at P20 (Fig. 1B). These results suggested that the long period of hair graying is due to the mixture of partly pigmented (old) and unpigmented (new) hair shafts (Supplemental Fig. S1B,C) rather than the continuous production of new gray hairs. To confirm this, gray *Scf^flox/gfp^; Krox20Cre* mice were depilated at 2 mo to remove all hairs, including old hairs, and stimulate new hair regeneration; as expected, their new hairs showed a complete loss of pigmentation (Fig. 1G). Most importantly, the fact that *Scf^flox/gfp^; Krox20Cre* mice underwent a complete loss of hair pigmentation suggested that *Krox20* lineage cells are the main source of SCF for follicular melanocytes to produce hair pigment.

### Hair pigmentation is dependent on SCF expression by epithelial keratinocytes

In order to identify the cell type that produces SCF in the skin, thereby regulating hair pigmentation and being responsible for the hair hypopigmentation in *Scf^flox/gfp^; Krox20Cre* mice, we examined the hair color phenotype of SCF ablation in different cell lineages in the skin by using several cell type-specific *Cre* mouse lines, including *DhhCre* (Schwann cell), *PLPCre*^*ERT2*^ (Schwann cell), *TyrCre*^*ERT2*^ (melanocyte), and *K14Cre* (keratinocyte). In addition, *CMVCre*^*ERT*^ (all cells) was included as a control for the inducible *Cre* systems. The *R26-LacZ* reporter was introduced into all of the above conditional knockouts to validate *Cre* expression specificity and efficiency.

**Figure 2.**
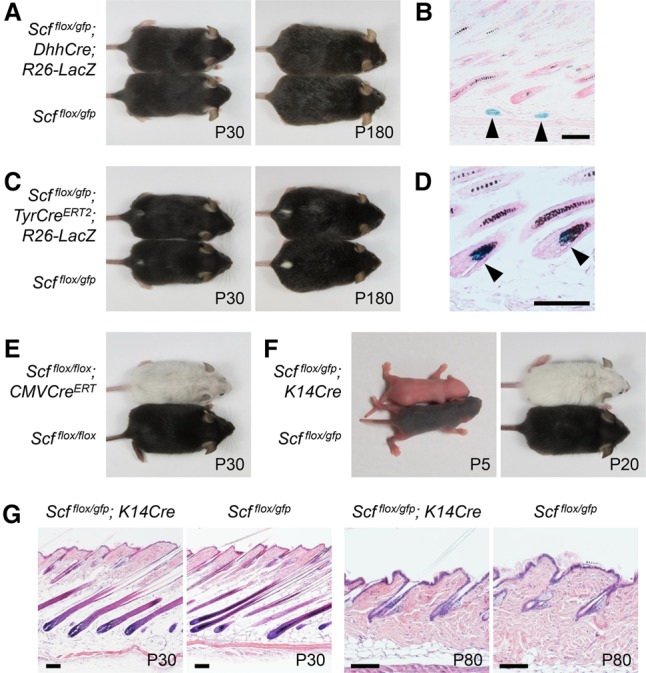
Hair pigmentation is dependent on SCF expression by epithelial keratinocytes. (*A*) Depletion of SCF in Schwann cells does not affect hair pigmentation. *Scf^flox/gfp^; DhhCre* mice exhibited normal hair pigmentation. *n* = 8. (*B*) LacZ staining revealed that *Dhh* lineage cells were present in subcutaneous nerves (arrowheads). (*C*) Depletion of SCF in melanocytes does not affect hair pigmentation. *Scf*^*flox/gfp*^; *TyrCre*^*ERT2*^ mice were induced by 4-hydroxytamoxifen at P0 and displayed normal hair pigmentation. *n* = 8. (*D*) LacZ staining revealed that *Tyr* lineage cells were present in matrix melanocytes (arrowheads). (*E*) *Scf*^*flox/flox*^; *CMVCre*^*ERT*^ mice were induced by 4-hydroxytamoxifen as in *C* to ablate SCF expression in all cells. These mice exhibited mostly hypopigmented hairs, and this lasted throughout their lives (*n* = 5), validating an optimal 4-hydroxytamoxifen induction in *C*. (*F*) Depletion of SCF in epithelial cells completely abolishes hair pigmentation. *Scf*^*flox/gfp*^; *K14Cre* mice had no hair pigmentation since birth, and this phenotype lasted throughout their lives. *n* = 8. (*G*) SCF expression in epithelial cells appears to regulate only hair pigmentation. In hematoxylin and eosin staining, *Scf*^*flox/gfp*^; *K14Cre* mice displayed skin structure and hair cycling similar to those of littermate controls. Nuclei were stained with nuclear Fast Red in *B* and *D*. Bar, 100 µm.

In mice lacking SCF in Schwann cells (*Scf^flox/gfp^; DhhCre*), we did not observe any hair color changes for up to 6 mo (Fig. 2A). LacZ staining confirmed the specific *DhhCre* expression in Schwann cells in cutaneous nerves (Fig. 2B). Similar results were observed in *Scf*^*flox/gfp*^; *PLPCre*^*ERT2*^ mice (Supplemental Fig. S2A). These results revealed that hair pigmentation does not depend on SCF expression in cells of Schwann cell lineages. Likewise, mice with SCF depletion in melanocytes (*Scf*^*flox/gfp*^; *TyrCre*^*ERT2*^) had no phenotype in hair color (Fig. 2C) after validation of Cre expression in the hair matrix melanocytes (Fig. 2D). Importantly, this result ruled out the possibility of melanocyte cell-autonomous SCF contributions to hair pigmentation.

In *Scf*^*flox/gfp*^; *PLPCre*^*ERT2*^ and *Scf*^*flox/gfp*^; *TyrCre*^*ERT2*^ mice, we activated *Cre* expression by injecting 4-hydroxytamoxifen at P0. To be certain that this timing was suitable for examining the requirement for SCF in hair pigmentation, we generated *Scf*^*flox/flox*^; *CMVCre*^*ERT*^ mice as a control to ablate *Scf* ubiquitously by the same 4-hydroxytamoxifen induction condition. These mice lost their hair pigmentation nearly completely after the use of 4-hydroxytamoxifen (Fig. 2E), validating the *Cre* induction conditions. Similar results were also observed when these Cre expressions were induced in adult mice (Supplemental Fig. S2D–G).

Finally, we generated *Scf^flox/gfp^; K14Cre* mice to examine the phenotype after SCF ablation in keratinocytes. Strikingly, depletion of SCF in keratinocytes resulted in mice displaying a complete absence of hair pigmentation, beginning at birth (Fig. 2F), and this phenotype was limited to hair pigmentation without affecting the development or growth of hair and the epidermis (Fig. 2G). This result demonstrated the critical contribution of SCF from epithelial keratinocytes to hair pigmentation. This finding also supports the non-cell-autonomous SCF signaling model for hair pigmentation.

In addition, albeit with different timing of onset, aged *Scf^flox/gfp^; Krox20Cre* mice phenocopied *Scf^flox/gfp^; K14Cre* mice by having a complete absence of hair pigmentation (Figs. 1A, 2F), suggesting that KROX20-expressing cells originate from *K14* lineage epithelial keratinocytes and that KROX20 expression in the HF starts later in development.

### Dynamic localization of *Krox20* lineage cells during HF morphogenesis

**Figure 3.**
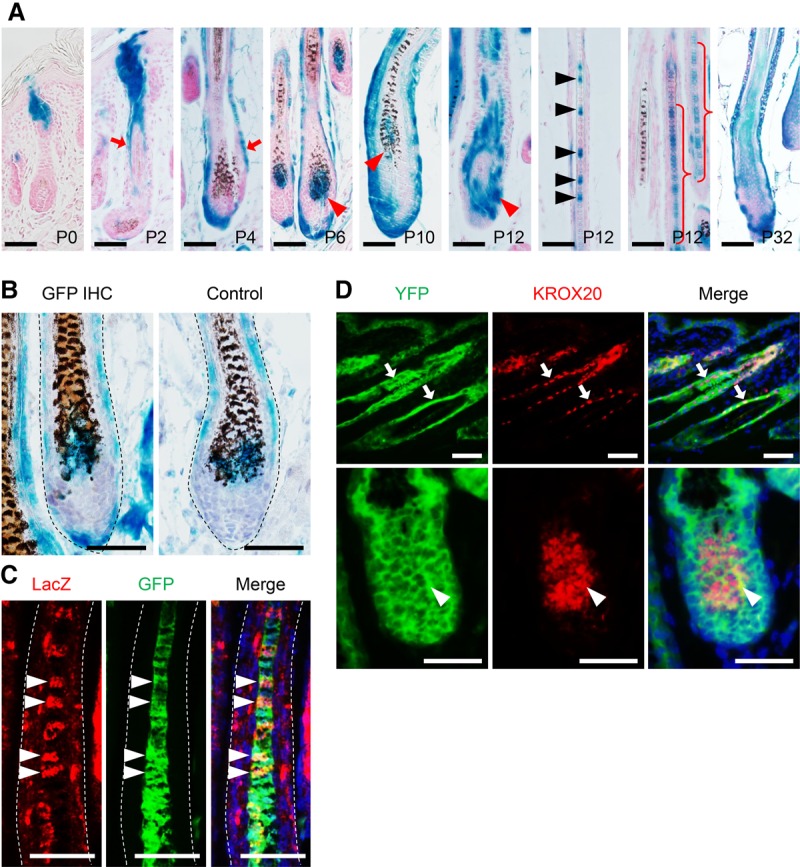
Dynamic localization of *Krox20* lineage cells during HF morphogenesis. (*A*) Lineage tracing of Krox20 by LacZ in the skin of postnatal *Scf^flox/gfp^; Krox20Cre; R26-LacZ* mice revealed a dynamic localization of *Krox20* lineage cells during HF morphogenesis. At P0, LacZ was detected only in the infundibulum and bulge of HFs. They then underwent an expansion along outer root sheath (arrows). Beginning around P6–P10, LacZ was also detected in the hair matrix (red arrowheads). Importantly, at P12, LacZ was detected in the hair shaft (black arrowheads and curly brackets), demonstrating that *Krox20* lineage traced the matrix progenitor cells giving rise to hair shafts. Note that the P12 matrix and hair shaft are absent of pigment. *Krox20* lineage also marks the same population of cells in the subsequent hair cycle at P32. (*B*) In P6 *Scf^flox/gfp^; Krox20Cre; R26-LacZ* HFs, *Krox20* lineage cells (marked by LacZ) were colocalized with SCF-expressing cells (marked by GFP) in the hair matrix. (*C*) In P12 *Scf^flox/gfp^; Krox20Cre; R26-LacZ* HFs, *Krox20* lineage cells (marked by LacZ) were colocalized with SCF-expressing cells (marked by GFP) in the hair shaft (arrowheads). (*D*) In P12 *K14Cre; R26-YFP* HFs, KROX20 is detected in the *K14* lineage epithelial cells in the outer root sheath (arrows) and the hair matrix (arrowheads). Nuclei were stained with nuclear Fast Red in *A*, hematoxylin in *B*, and DAPI in *C* and *D*. Bar, 50 µm.

To characterize the late onset of hair hypopigmentation in *Scf^flox/gfp^; Krox20Cre* mice, we explored *Krox20* lineage cells in skin at different HF developmental stages by crossing the *Krox20Cre* mice with *R26-LacZ*. Interestingly, we found that LacZ^+^ cells were detected only as a small population of cells located at the infundibulum and bulge of the HF at P0. At P2, LacZ^+^ cells occupied the entire upper portion of the HF. However, as the HF developed, they expanded in numbers along the outer root sheath and then colonized the matrix, beginning at about P6–P10. We analyzed at least three mice from each age and observed a consistent recombination pattern (Fig. 3A). At the final stage of differentiation, hair matrix keratinocytes undergo a unique type of programmed cell death that is marked by systematic destruction of the nucleus. These keratinized anucleate cells become enveloped by specialized and highly cross-linked membranes that eventually form the building blocks of the hair shaft. Strikingly, in P12 skin, we also observed LacZ activity within the hair shafts (Fig. 3A). This is direct evidence demonstrating that *Krox20* lineage labels hair progenitor cells that give rise directly to the hair shafts. Importantly, considering the timing of the first appearance of LacZ^+^ cells in the hair matrix, *Krox20* lineage appears to mark only a subsequent wave of hair progenitors postnatally. This observation corresponds with the late onset of hair hypopigmentation in the *Scf^flox/gfp^; Krox20Cre* mice (Fig. 1A; Supplemental Fig. S1A). It also revealed distinct developmental waves of hair shaft progenitor cells and, importantly, suggested that hair pigmentation is regulated by SCF expression in hair shaft progenitor cells.

We therefore examined SCF expression in *Krox20* lineage cells by *Scf* promoter-driven GFP (*Scf*^*gfp*^). Our data showed that SCF-expressing and *Krox20* lineage cells were colocalized at the hair matrix at P6 (the time at which *Krox20* lineage cells begin to colonize in the matrix) (Fig. 3B) and at the hair shaft at P12 (the time at which *Krox20* lineage cells build the hair shaft) (Fig. 3C). These results support the model in which SCF expression in hair shaft progenitor cells controls hair pigmentation.

Our lineage tracing of *Krox20* revealed a new identity of epithelial cells giving rise to the hair shaft (Fig. 3A). To determine the developmental origin of KROX20-expressing cells in the HFs, we generated *K14Cre; R26-YFP* mice to genetically label all *K14* lineage epithelial keratinocytes early in development with YFP. We subsequently performed immunofluorescence staining against KROX20 and observed that KROX20 is expressed in a subpopulation of the of *K14* lineage cells in the HFs (Fig. 3D), indicating that that HF KROX20^+^ cells are differentially derived from a K14^+^ keratinocyte lineage. The detection of KROX20 protein in the outer root sheath and matrix demonstrates that KROX20 is expressed actively during hair development. Taken together with the findings that *Scf* depletion in Schwann cells does not cause hair hypopigmentation (Fig. 2A; Supplemental Fig. S2A,D), these results distinguish the origin of KROX20^+^ HF cells (epithelial derivative) from KROX20^+^ Schwann cells (neural crest derivative) during skin development.

Because hair shaft progenitor cells are differentiated from HF stem cells in the bulge and because their activation is associated with hair cycle, we therefore tested the hypothesis that depletion of SCF in HF stem cells will result in hair hypopigmentation in a hair cycle-dependent manner. To address this hypothesis, we used the inducible *K14Cre*^*ERT*^ mouse line (Vasioukhin et al. 1999) to examine the effects of *Scf* gene ablation in HF stem cells. The inductions of SCF ablation in *Scf*^*flox/gfp*^; *K14Cre*^*ERT*^ mice were performed at different HF growth stages. *Scf*^*flox/gfp*^; *K14Cre*^*ERT*^ mice with induction of SCF ablation at P0 (in anagen, when some hair shaft progenitor cells have already formed) showed normal hair pigmentation initially; however, their coat color turned gray after subsequent anagen (Supplemental Fig. S3A), phenocopying the *Scf^flox/gfp^; Krox20Cre* mice. On the other hand, *Scf*^*flox/gfp*^; *K14Cre*^*ERT*^ mice with induction of SCF loss and depilation together at P90 (in telogen, when hair shaft progenitor cells have not yet formed) showed an immediate depigmented coat in the newly regenerated hairs (Supplemental Fig. S3B). These results revealed that induction of SCF loss in HF stem cells at different HF developmental stages can result in different patterns of hair color changes. Ablation of SCF in HF stem cells at telogen depleted SCF in all subsequent differentiating HF cells; therefore, all hair shaft progenitor cells acquired SCF ablation and gave rise to hypopigmented hairs immediately. In contrast, induction of SCF loss in HF stem cells at anagen depleted SCF only in newly differentiated hair shaft progenitor cells but not those differentiated before the induction, explaining the normally pigmented hairs followed by hypopigmented hairs (Supplemental Fig. S3E). These results supported our conclusion that the active expression of SCF in hair shaft progenitor cells is critical for hair pigmentation.

### The hair matrix is the SCF-dependent niche for hair pigmentation

In *Scf^flox/gfp^; K14Cre* mice, the complete loss of hair pigmentation is caused by *Scf* deletion in *K14* lineage cells. We therefore hypothesized that this depigmentation could be rescued by ectopic *Scf* expression under a *K14* promoter. To test this hypothesis, we used a mouse transgene with a *K14* promoter-driven membrane-bound form of *Scf* (*K14-Scf*), which produces a dominant phenotype of epidermal hyperpigmentation (Kunisada et al. 1998a). Given that a mouse mutant expressing only a soluble form of SCF (*Sl*^*d*^/*Sl*^*d*^) has no skin or hair pigmentation (Copeland et al. 1990; Brannan et al. 1991), we reasoned that hair pigmentation is dependent on membrane-bound SCF and thus that the *K14-Scf* transgene should be a suitable tool to address the requirement of SCF for hair pigmentation.

**Figure 4.**
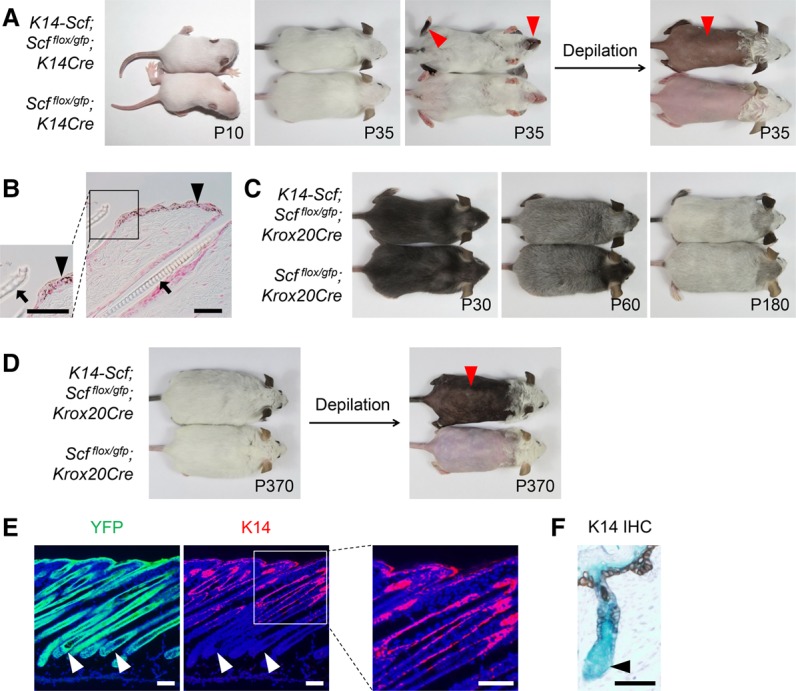
The hair matrix is the SCF-dependent niche for hair pigmentation. (*A*) Ectopic SCF expression by *K14* promoter-driven *Scf* (*K14-Scf*) does not rescue hair hypopigmentation. *K14-Scf; Scf^flox/gfp^; K14Cre* mice displayed white hairs and dark skin (arrowheads). *n* = 8. (*B*) Skin histology in *K14-Scf; Scf^flox/gfp^; K14Cre* mice revealed epidermal hyperpigmentation (arrowheads) and unpigmented hair shafts (arrows). (*C*) *K14-Scf* does not rescue hair graying. *K14-Scf; Scf^flox/gfp^; Krox20Cre* mice underwent hair graying similar to *Krox20Cre; Scf*^*flox/gfp*^ controls. *n* = 8. (*D*) *K14-Scf* transgene remained active along aging, as indicated by the permanent skin hyperpigmentation (arrowhead) in 1-yr-old mice. *n* = 3. (*E*) Hair matrix cells differentiate from *K14* lineage cells but no longer express K14. K14 immunostaining in *K14Cre; R26-YFP* skin from P10 mice revealed that K14-expressing cells are restricted in the interfollicular epithelium and the top portion of HFs. Importantly, HF matrix cells (arrowheads) do not express K14, although they differentiated from *K14* lineage cells (marked by YFP). Nuclei were stained with DAPI. (*F*) Using a similar approach as in *D*, in P0 skin, K14 immunohistochemical staining marked the interfollicular epithelium and the top portion of HFs but not HF matrix cells (arrowhead), whereas *K14* lineage (marked by LacZ) traced to the entire skin and HF epithelium. Bars: *B*,*E*, 50 µm; *D*, 100 µm.

Surprisingly, the *K14-Scf; Scf^flox/gfp^; K14Cre* mice displayed white hair with dark skin (Fig. 4A,B), indicating that even though *Scf* is expressed under a K14 promoter, *K14-Scf* was unable to rescue the hair depigmentation in *Scf^flox/gfp^; K14Cre* mice. Likewise, we observed that *K14-Scf* did not rescue the hair-graying progress in *Scf^flox/gfp^; Krox20Cre* mice. The *K14-Scf; Scf^flox/gfp^; Krox20Cre* mice underwent hair graying identical to that of *Scf^flox/gfp^; Krox20Cre* control mice (Fig. 4C), although the *K14-Scf* transgene appeared to remain active over time (Fig. 4D). These findings are similar to earlier experiments in which the *K14-Scf* transgene was unable to rescue hair hypopigmentation in *Sl*^*d*^/*Sl*^*d*^ mice (Yoshida et al. 2001) or KIT-neutralizing antibody ACK2-treated mice (Nishimura et al. 2002).

To explore the underlying mechanism by which ectopic expression of *K14-Scf* failed to rescue the *Scf* loss-induced hair hypopigmentation, we reasoned that perhaps the K14 promoter activity is turned off in the KROX20^+^ hair shaft progenitor cells in the matrix, and therefore the lack of SCF expression had regulated hair pigmentation. To address this question, we lineage-traced the cells with “*Scf* ablation” by *R26-YFP* (as a marker for *K14Cre* lineage) and identified the cells with “*Scf* reconstitution” by K14 immunostaining (as a marker for *K14* promoter) in the skin from *K14Cre; R26-YFP* mice. Intriguingly, YFP was detected in the interfollicular epithelium and the entire HF (except dermal papillae), whereas K14 was detected only in the interfollicular epithelium and only the top portions of anagen HF (Fig. 4E); similar results were obtained from *K14Cre; R26-LacZ* mice (Fig. 4F). This expression pattern matches previous studies on *K14Cre* lineage tracing (Huelsken et al. 2001) and K14 expression (Coulombe et al. 1989). Most importantly, these data demonstrate that hair pigmentation is tightly dependent on SCF from the *Krox20* lineage cells in the hair matrix of HFs that are differentiated from *K14* lineage but no longer express K14.

**Figure 5.**
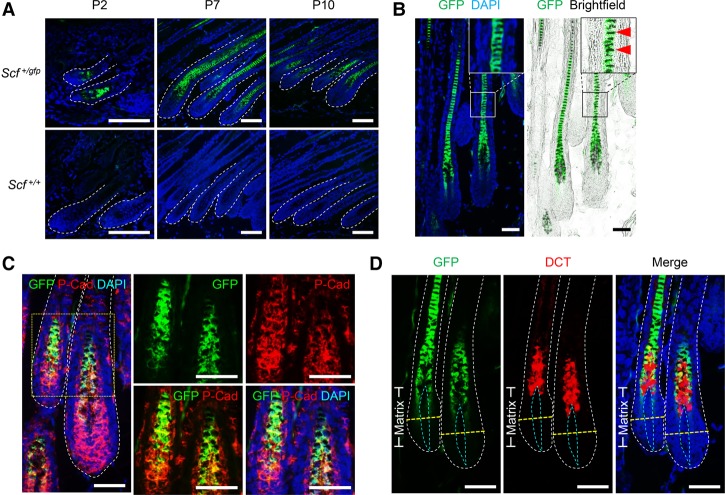
SCF is expressed in hair shaft progenitor cells. (*A*) Scf promoter-driven GFP (*Scf*^*gfp*^) showed that SCF is expressed in the upper HF matrix and hair shaft throughout anagen. (*B*) SCF is expressed in melanin-containing cells (arrowheads) in the HF in P10 *Scf*^+/*gfp*^ skin. (*C*) SCF is expressed in a subpopulation of P-cadherin^+^ HF matrix cells and the hair shaft in P9 *Scf*^+/*gfp*^ skin. (*D*) DCT^+^ melanocytes are tightly adjacent to SCF-expressing cells in the upper HF matrix in P10 *Scf*^+/*gfp*^ skin. (Yellow dashed line) Line of Auber (an imaginary line drawn across the widest region of the hair bulb); (blue dashed line) dermal papilla. Nuclei were stained with DAPI. Bars: *A*, 100 µm; *B*–*D*, 50 µm

To better characterize the expression of SCF during HF development, we then took advantage of another marker, GFP, to locate the SCF-expressing cells by using the *Scf*^*gfp*^ mice. We found that GFP^+^ cells are predominantly localized in the upper hair matrix and hair shaft (but not the bulge or outer root sheath area) throughout the anagen stage (Fig. 5A), pointing to the hair shaft progenitor cells as a unique cell type expressing SCF during the HF growth stage. This observation was supported by the inclusion of melanin pigment (Fig. 5B) and the expression of P-cadherin (Fig. 5C) in these SCF-expressing cells. P-cadherin is an epithelial cell–cell adhesion molecule and, in HFs, marks the outer root sheath, inner matrix, and hair shaft (Samuelov et al. 2012). As the hair matrix is also colonized by melanocytes, we therefore determined the location of mature melanocytes by immunostaining of dopachrome tautomerase (DCT). DCT is an enzyme participating in melanin biogenesis and, in HFs, marks mature melanocytes in the hair matrix predominantly and a small number of melanocyte stem cells in the bulge (Nishimura et al. 2005). DCT^+^ melanocytes are tightly adjacent to the SCF-expressing cells in the matrix (Fig. 5D); this result is in agreement with the identification of matrix SCF-expressing cells as the hair shaft progenitor cells. Taken together, we concluded that hair pigmentation is controlled exclusively by SCF on hair shaft progenitor cells, which regulates melanocytes in the HF matrix in a non-cell-autonomous fashion.

### SCF in the matrix is crucial for the maintenance of mature melanocytes

We showed that depletion of SCF in the hair shaft progenitor cells results in loss of hair pigmentation. In addition, melanocytes are the specialized melanin-producing cells as well as the predominant KIT-expressing cells in the HF (Lin and Fisher 2007). Therefore, melanocytes mediate SCF signaling to hair pigmentation, and SCF is necessary for melanocytic activity and hair pigmentation. Thus, we next addressed the fate of follicular melanocytes in response to the loss of SCF in the hair shaft progenitor cells.

**Figure 6.**
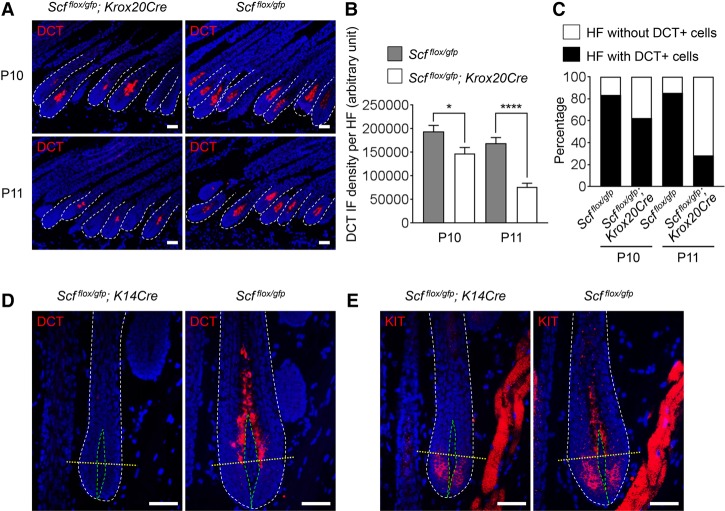
SCF is required for the maintenance of mature melanocytes. (*A*) Loss of matrix DCT^+^ melanocytes in *Scf^flox/gfp^; Krox20Cre* mice. DCT immunostaining revealed that the number of matrix DCT^+^ melanocytes decreased rapidly right before hair lost pigmentation in *Scf^flox/gfp^; Krox20Cre* mice. (*B*) Quantification and statistical analysis of *A* revealed a significant and rapid decrease of matrix DCT staining intensity in *Scf^flox/gfp^; Krox20Cre* mice during P10–P11. Data are mean ± SEM from *n* ≥ 53 HFs in each group. (****) *P* < 0.0001; (*) *P* < 0.05. (*C*) Quantification and statistical analysis of *A* revealed increased numbers of hair matrices without DCT in *Scf^flox/gfp^; Krox20Cre* mice during P10–P11. *n* ≥ 53 HFs per group. (*D*) DCT immunostaining revealed that *Scf^flox/gfp^; K14Cre* mouse HFs were completely absent of matrix DCT^+^ mature melanocytes. (*E*) KIT immunostaining revealed that *Scf^flox/gfp^; K14Cre* mouse HFs lost KIT^+^ melanocytes above the Line of Auber (differentiated mature melanocytes); however, KIT^+^ melanocytes still detected below the Line of Auber. Nuclei were stained with DAPI. (Yellow dashed line) Line of Auber; (green dashed line) dermal papillae. Bar, 50 µm.

The *Scf^flox/gfp^; Krox20Cre* mice began to lose hair pigmentation at P11–P13 due to SCF loss in their hair shaft progenitor cells (Fig. 1D–F). We took advantage of this model to address the fate of melanocytes during SCF loss. As hair shaft progenitor cells are tightly adjacent to melanogenic melanocytes in the upper HF matrix (Fig. 5D), we thus hypothesized these mature melanocytes to be the first affected cells. Indeed, DCT immunostaining revealed a significant loss of DCT^+^ differentiated mature melanocytes in the *Scf^flox/gfp^; Krox20Cre* mouse HF at P10 (*P* < 0.05) and P11 (*P* < 0.0001) (Fig. 6A–C). This result demonstrated a critical role of SCF in the maintenance of mature melanocytes.

Furthermore, we explored the fate of DCT^+^ melanocytes in another mouse model: *Scf^flox/gfp^; K14Cre*. These mice had a complete loss of hair pigmentation throughout their lifetimes (Fig. 2F). Therefore, it was not surprising that DCT^+^ melanocytes were not detected in their hair matrices (Fig. 6D). The HF melanocytes have been characterized previously to express KIT, which is the receptor for SCF (Lennartsson and Ronnstrand 2012). Melanocytes in the upper and lower follicular matrix compartments can be separated by the Line of Auber (an imaginary line drawn across the widest region of the hair bulb) (Peters et al. 2002). Melanocytes in the lower matrix are relatively undifferentiated and nonmelanogenic. However, differentiated mature melanocytes reside in the upper HF matrix and produce melanin. We further characterized the extent to which SCF can affect these two melanocyte populations by utilization of this other melanocyte marker: KIT. KIT^+^ melanocytes were present in both lower and upper HF matrices in the control skin. However, in the skin from *Scf^flox/gfp^; K14Cre* mice, KIT^+^ melanocytes were detected only in the lower matrix but were completely absent in the upper HF matrix (Fig. 6E). This finding demonstratesthat loss of SCF in the hair shaft progenitor cells influences only the mature melanocytes, highlighting the upper HF matrix as a critical niche where non-cell-autonomous SCF/KIT signaling regulates hair pigmentation. It also points to the critical role of follicular epithelial SCF in controlling the fate of melanocytes in their final maturation without affecting their previous differentiation and migration.

### HF *Krox20* lineage cells are critical for hair development

**Figure 7.**
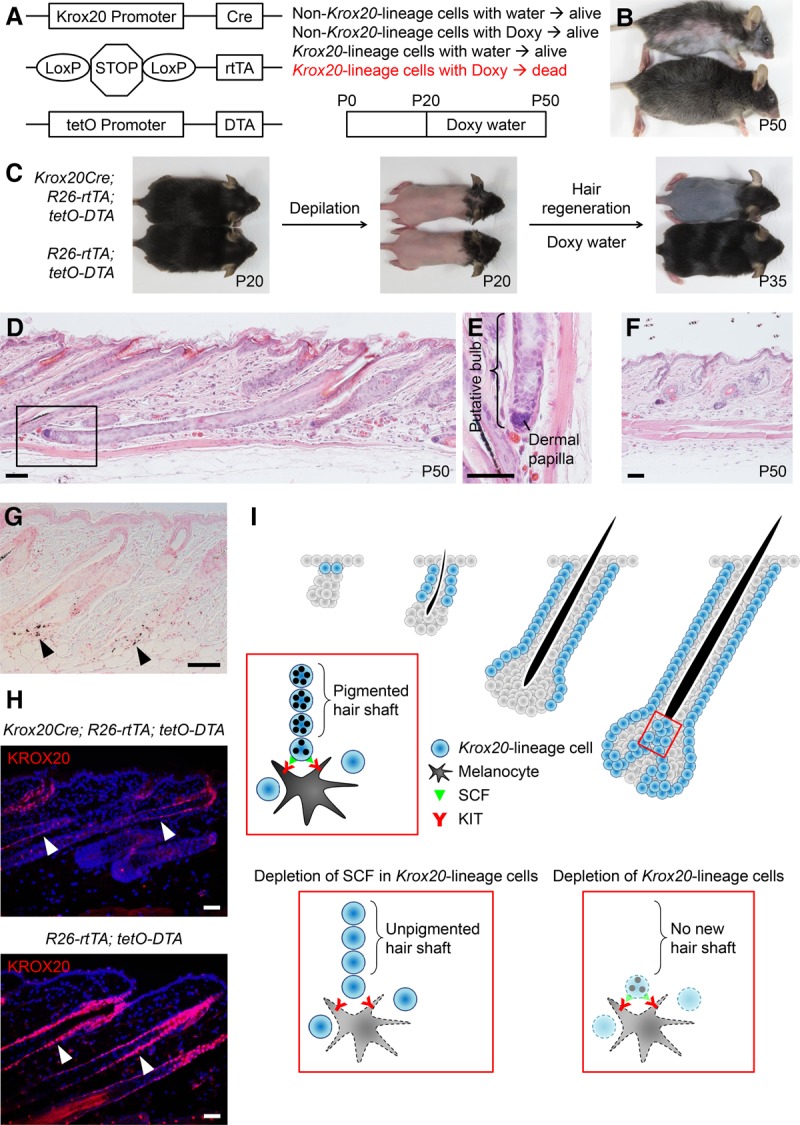
HF *Krox20* lineage cells are critical for hair development. (*A*) Schematic illustration of the doxycycline-inducible mouse model to deplete *Krox20* lineage cells in vivo. *Krox20Cre; R26-rtTA; tetO-DTA* allows the killing of *Krox20* lineage cells by DTA in mice upon doxycycline treatment. (*B*) *Krox20Cre; R26-rtTA; tetO-DTA* (*top*) and littermate control (*bottom*) mice were treated with doxycycline from P20 to P50. Remarkable hair loss was observed in *Krox20Cre; R26-rtTA; tetO-DTA* mice at P50. *n* = 8. (*C*) *Krox20Cre; R26-rtTA; tetO-DTA* mice were depilated at P20 followed by doxycycline treatment from P20 to P35; notably impaired new hair growth was observed. *n* = 3. (*D*) The HFs in P50 *Krox20Cre; R26-rtTA; tetO-DTA* mice were arrested in anagen with an atypical HF structure composed of elongated HFs and shrunken hair bulbs. (*E*) A close-up view of *D* to emphasize the aberrant hair bulb structure. (*F*) The HFs in P50 *R26-rtTA; tetO-DTA* control mice were rested in telogen. (*G*) Ectopic melanin deposition (arrowheads) around HFs was noticed in *Krox20Cre; R26-rtTA; tetO-DTA* mice. (*H*) KROX20 immunostaining confirmed a substantial reduction of KROX20^+^ cells in *Krox20Cre; R26-rtTA; tetO-DTA* mice after doxycycline treatment. Nuclei were stained with DAPI. (*I*) Schematic illustration of the roles of SCF and *Krox20* lineage cells in hair pigmentation and development. Lineage tracing of *Krox20* lineage cells revealed a dynamic expansion toward the direction of hair-producing cells during HF morphogenesis in the upper HF matrix (red box). Hair progenitor cells sustain differentiated melanocytes through non-cell-autonomous SCF/KIT signaling. Depletion of SCF in hair progenitor cells results in loss of hair pigmentation. Depletion of *Krox20* lineage cells, the cells that differentiate into hair-producing cells, results in impaired hair regeneration. Bar, 50 µm.

Based on our work, we concluded that the transcription factor KROX20 marks a lineage of resident hair shaft progenitor cells in the follicular matrix. Therefore, we hypothesized that *Krox20* lineage cells are indispensible for hair growth. To test this hypothesis, we generated a doxycycline-inducible mouse model (*Krox20Cre; R26-rtTA; tetO-DTA*) to deplete *Krox20* lineage cells in vivo. The *R26-rtTA* expresses reverse tetracycline-controlled transactivator (rtTA) (Belteki et al. 2005) in the *Krox20* lineage cells. The *tetO-DTA* expresses diphtheria toxin A (DTA) (Lee et al. 1998) to eliminate cells with rtTA expression (*Krox20* lineage cells in this case) upon doxycycline induction (Fig. 7A). We used this model to examine the effects of *Krox20* lineage cell depletion during the second anagen phase (starting at about P23–P24). Mice were fed with doxycycline water starting at P20. We observed signs of hair loss (rough coat) starting at starting P40, and they had significant hair loss by P50 (Fig. 7B). As expected, these mice also displayed weak muscle tone (data not shown), which is likely associated with the peripheral nerve demyelination caused by KROX20^+^ cell depletion (Decker et al. 2006). To determine whether this hair loss, induced by *Krox20* lineage cell depletion, was caused by impaired hair growth or increased hair shedding, we examined the effects of depilation on new hair growth. Mice were depilated to induce new hair growth at P20. At the same time, they were started on doxycycline water to deplete *Krox20* lineage cells in vivo. Within 15 d, control mice (*R26-rtTA; tetO-DTA*) regrew their entire hair coat. On the other hand, *Krox20Cre; R26-rtTA; tetO-DTA* mice had a complete arrest of new hair generation (Fig. 7C), demonstrating the critical role of *Krox20* lineage cells in hair development.

To characterize further the role of *Krox20* lineage cells in HF development, we examined the skin of *Krox20Cre; R26-rtTA; tetO-DTA* mice after doxycycline treatment between P20 and P50 to assess the impact of *Krox20* lineage cell depletion on hair regeneration. We found that *Krox20* lineage cell-depleted HFs were arrested at anagen (Fig. 7D), whereas the control HFs completed the anagen phase and rested at telogen (Fig. 7F). Importantly, in contrast to normal anagen HFs, *Krox20* lineage cell-depleted HFs had shrunken hair bulbs, which were located near to where the hair shaft progenitor cells had resided previously (Fig. 7E). This atypical anagen phase, with missing stereotypic bulb structure (the reservoir of matrix cells), explained their inability to grow new hair. We also noticed that the *Krox20* lineage cell-depleted skin exhibited ectopic melanin deposition around the HFs (Fig. 7G); this is likely contributed by the death of *Krox20* lineage cells after obtaining the melanin from melanocytes, as we started the doxycycline treatment on the same day of depilation, and therefore it will take some time after the doxycycline treatment for the diphtheria toxin A to delete the *Krox20* lineage cells. The appropriateness of this system was validated by faithful depletion of KROX20 cells in vivo after doxycycline induction (Fig. 7H). Taken together, these analyses confirm the critical role of *Krox20* lineage cells in hair development as the cells that produce hair directly.

## Discussion

We identified that the transcription factor KROX20 marks a cell lineage differentiating toward the hair shaft and that SCF in these hair shaft progenitor cells acts as a critical intrinsic rheostat of hair pigmentation by managing mature melanocyte in the upper HF matrix. Ablation of *Scf* in *Krox20* lineage cells consequently leads to a complete absence of hair pigmentation, showing an indispensible non-cell-autonomous SCF contribution to melanocytes and that *Krox20* lineage cells are the main source of SCF for follicular mature melanocytes to produce hair pigment. The completion of hair regeneration is dependent on the presence of HF *Krox20* lineage cells as the cells giving rise directly to hair shafts (Fig. 7I).

SCF production in keratinocytes appears solely to regulate the generation of hair pigmentation by melanocytes because no skin or hair developmental defect was observed in the *Scf^flox/gfp^; Krox20Cre* or *Scf^flox/gfp^; K14Cre* mice (Figs. 1D, 2G). The critical role of SCF/KIT signaling in hair pigmentation has long been recognized (Copeland et al. 1990; Reith et al. 1990; Okura et al. 1995); however, this is the first report to identify *Krox20* lineage hair shaft progenitor cells definitively as the source of SCF to control this process. This discovery could lead to additional studies to characterize their contributions to other signaling events that are critical for melanocyte development, including the effects of Notch (Schouwey et al. 2007), TGFβ (Nishimura et al. 2010), and β-catenin (Rabbani et al. 2011). During the development of melanocytes, the neural crest stem cells generate melanoblasts that subsequently become melanocyte stem cells. Although melanocyte stem cells in the bulge do not require SCF, their differentiation into mature melanocytes is SCF-dependent (Nishimura et al. 2002), consistent with our finding that loss of SCF in hair shaft progenitor cells targets only the mature melanocytes in the matrix and not the undifferentiated melanocyte precursors.

While dissecting the hair-graying phenotype of *Scf^flox/gfp^; Krox20Cre* mice, we surprisingly discovered that this hair color change is associated with distinct stages in the cells committed to hair production. *Krox20* lineage marks the hair shaft progenitor cells only after P6–P10 (Fig. 3A), which demonstrates the presence of a second wave of hair shaft progenitor cells and explains the late onset of hair hypopigmentation and eventual complete depigmentation in *Scf^flox/gfp^; Krox20Cre* mice. Interestingly, late onset of the hair hypopigmentation phenotype has also been observed in other genetically modified mice. *Bcl2*^−/−^ mice (Veis et al. 1993) and *Notch1^flox/+^; Notch2^flox/flox^; TyrCre* mice (Schouwey et al. 2007) exhibit hair graying that is similar to that of the *Scf^flox/gfp^; Krox20Cre* mice. Loss of BCL2-dependent melanocyte stem cells beginning from P8 has been identified to cause the hair graying in *Bcl2*^−/−^ mice (McGill et al. 2002; Nishimura et al. 2005). On the other hand, β*-cat^flox/−^; K14Cre* mice (Huelsken et al. 2001) display late-onset hair loss. They develop hairs after birth, but most of these hairs are lost as they grow into adulthood. Taken together, these late-onset phenotypes might be related to our two-wave hair growth model. Furthermore, this model could shed light on the classification of embryonic and adult stem cells that are committed to the same type of tissue generation.

KROX20 is best known as a transcription factor for Schwann cells during differentiation from promyelinating to myelinating states (Topilko et al. 1994), which takes place primarily during early postnatal development (Zorick et al. 1999). This phenomenon is similar to the expansion of HF *Krox20* lineage cells during hair morphogenesis (Fig. 3A). A recent study has revealed that peripheral nerve KROX20 induction is regulated by the Lin28/*Let-7* axis (Gokbuget et al. 2015). Lin28 is a conserved RNA-binding protein that is expressed highly in embryonic stem cells. It is also diminished sharply after birth; this change results in the induction of *Let-7* microRNA (Shyh-Chang and Daley 2013). Interestingly, *Lin28a* transgenic mice exhibit a thickened hair coat due to prolonged anagen (Shyh-Chang et al. 2013). These results suggest that KROX20 could be a downstream regulator for Lin28/*Let-7*-mediated perinatal development in both nerves and skin.

The bone morphogenetic protein (BMP) and Wnt/β-catenin signaling plays a central role in epidermal homeostasis and HF development (Blanpain and Fuchs 2009; Solanas and Benitah 2013; Plikus and Chuong 2014). Mice with *K5* promoter-driven BMP antagonist noggin (*K5-noggin*) are characterized by enlarged anagen HFs with thickened hair shafts; this feature is associated with hyperproliferative hair matrix cells with a sixfold decrease in *Krox20* expression (Sharov et al. 2006). Similarly, *K14-noggin* mice have a faster response to hair regeneration (Plikus et al. 2008). Moreover, Wnt activation leads to up-regulation of KROX20, and this induction can be synergized by BMP inhibition with noggin (Saint-Jeannet et al. 1997; Baker et al. 1999). Taken together with our findings, the functional prediction for KROX20 in HF development could be a downstream effector of Wnt/β-catenin and BMP signaling, thereby directing HF stem cell differentiation.

In conclusion, this study delineates the origin of SCF expression in the hair matrix progenitors as a niche for follicular mature melanocytes and that their SCF is indispensible for hair pigmentation. In addition, SCF expression in the matrix identifies immediate antecedents of hair shaft structural cells, and transcription factor KROX20 marks a sublineage of epithelial cells differentiating toward this hair shaft progenitor cells. Future studies to reconstitute SCF in the matrix niche will be interesting and will address whether hair hypopigmentation could be reversible. Furthermore, genetic ablation of *Krox20* and global profiling of KROX20 transcriptional targets will help to elucidate its biological roles in epidermal differentiation and HF development.

## Materials and methods

### Mice

Animal care and experiments were approved by the Institutional Animal Care and Use Committee at University of Texas Southwestern Medical Center. *Scf*^*flox*^ and *Scf*^*gfp*^ were generated in a previous report (Ding et al. 2012). *K14Cre* (Dassule et al. 2000), *K14Cre*^*ERT*^ (Vasioukhin et al. 1999), *TyrCre*^*ERT2*^ (Bosenberg et al. 2006), *R26-rtTA* (Belteki et al. 2005), and *tetO-DTA* (Lee et al. 1998) mice were purchased from the Jackson Laboratory. *K14-Scf* (Kunisada et al. 1998a) was kindly provided by Dr. John Harris (University of Massachusetts Medical School). *Krox20Cre* (Voiculescu et al. 2000), *DhhCre* (Skucas et al. 2013), *PLPCre*^*ERT2*^ (Leone et al. 2003), *CMVCre*^*ERT*^ (Hayashi and McMahon 2002), *R26-LacZ*, and *R26-YFP* were described previously (Le et al. 2009; Chen et al. 2014).

### Tamoxifen and 4-hydroxytamoxifen induction

Tamoxifen or 4-hydroxytamoxifen (Sigma-Aldrich) was administered to mice to induce *Cre*^*ERT*^ or *Cre*^*ERT2*^ expression. For induction in a newborn mouse, a single-dose treatment of 40 µg of 4-hydroxytamoxifen was injected subcutaneously. For induction in adult mice, 3 mg of tamoxifen was oral-gavaged daily for five consecutive days.

### Doxycycline induction

For the induction of *tetO-DTA*, mice were fed with water containing 2 mg/mL doxycycline (Sigma-Aldrich) and 5% sucrose.

### Mouse hair depilation

Mice were anesthetized by intraperitoneal injection of 100 µL (for a 25-g mouse) of a mixture of 30 mg/mL ketamine and 4 mg/mL xylazine solution. Mouse hair was trimmed by an electrical clipper. Depilatory cream (Nair) was applied gently on the area to be depilated and then wiped off with water-moistened gauzes to remove the hair.

### Histology analysis

For histology analysis, skin tissue was harvested and fixed in 10% formalin overnight followed by paraffin embedding. The tissue was then sectioned at 5-µm thickness. Hematoxylin and eosin staining was performed according to the manufacturer's protocol (StatLab).

### LacZ staining

For LacZ staining, mice were anesthetized by intraperitoneal injection of ketamine and xylazine as described above. Mice were then subjected to total body perfusion with 4% paraformaldehyde. Skin was harvested and fixed in 4% paraformaldehyde for 30 min followed by PBS rinses. LacZ staining was performed in 4-chloro-5-bromo-3-indolyl-β-galactoside (X-gal) solution (1 mg/mL X-gal, 4 mM potassium ferrocyanide, 4 mM potassium ferricyanide, 2 mM magnesium chloride in PBS) overnight at 37°C. X-gal-stained tissue was fixed in 10% formalin overnight and then subjected to paraffin embedding and tissue sectioning. Nuclei were counterstained with nuclear Fast Red.

### Fontana-Masson staining

Paraffin-embedded tissue sections were deparaffinized and rehydrated. Melanin staining was performed by microwaving tissue sections in ammoniacal silver working solution for 2 min. The ammoniacal silver stock solution was prepared by adding ammonium hydroxide to 10% silver nitrate until the solution precipitated and cleared again; the working solution was freshly prepared by mixing the stock solution with water at 1:3 ratio followed by filtering. Slides were then incubated with 0.1% gold chloride for 10 min and 5% sodium thiosulfate for 5 min with water rinses between each step. Nuclei were counterstained with nuclear Fast Red.

### Immunostaining

For immunostaining, frozen sections or paraffin sections after deparaffinization, rehydration, and antigen retrieval were used. The primary antibodies used in this study were P-cadherin (R&D Systems, AF761), DCT (PEP8h; gift from Dr. Vincent Hearing, National Institutes of Health) (Virador et al. 2001), K14 (biotin-labeled, Thermo, clone LL002), LacZ (Abcam, ab9361), GFP/YFP (Aves, 1020), Krox20 (Covance, PRB-236P), and KIT (Cell Signaling, 3074). For immunofluorescent staining, the primary antibodies were detected by secondary antibodies or streptavidin conjugated with Cy3 or Alexa fluor 488 (Jackson ImmunoResearch), and nuclei were counterstained with DAPI (Vector Laboratories). For immunohistochemical staining, the primary antibodies were detected by secondary antibodies or streptavidin conjugated with 3,3′ diaminobenzidine (Vector Laboratories).

### Statistical analysis

Data statistical analyses were performed by unpaired two-tailed Student's *t*-test (Prism7, GraphPad). Data are mean ± SEM. Significant differences were noted by asterisks (P < 0.05 [*], P < 0.01 [**],P < 0.001 [***], and P < 0.0001 [****]).

### Densitometry analysis

The quantification of HF melanin density and DCT immunofluorescence staining intensity was performed with ImageJ software (National Institutes of Health) in arbitrary units.

## Supplementary Material

Supplemental Material

## References

[LIAOGAD298703C1] Baker JC, Beddington RS, Harland RM. 1999 Wnt signaling in *Xenopus* embryos inhibits bmp4 expression and activates neural development. Genes Dev 13: 3149–3159. 10601040 10.1101/gad.13.23.3149 PMC317181

[LIAOGAD298703C2] Belteki G, Haigh J, Kabacs N, Haigh K, Sison K, Costantini F, Whitsett J, Quaggin SE, Nagy A. 2005 Conditional and inducible transgene expression in mice through the combinatorial use of Cre-mediated recombination and tetracycline induction. Nucleic Acids Res 33: e51. 15784609 10.1093/nar/gni051 PMC1069131

[LIAOGAD298703C3] Blanpain C, Fuchs E. 2009 Epidermal homeostasis: a balancing act of stem cells in the skin. Nat Rev Mol Cell Biol 10: 207–217. 19209183 10.1038/nrm2636 PMC2760218

[LIAOGAD298703C4] Blanpain C, Lowry WE, Geoghegan A, Polak L, Fuchs E. 2004 Self-renewal, multipotency, and the existence of two cell populations within an epithelial stem cell niche. Cell 118: 635–648. 15339667 10.1016/j.cell.2004.08.012

[LIAOGAD298703C5] Bosenberg M, Muthusamy V, Curley DP, Wang Z, Hobbs C, Nelson B, Nogueira C, Horner JW II, Depinho R, Chin L. 2006 Characterization of melanocyte-specific inducible Cre recombinase transgenic mice. Genesis 44: 262–267. 16676322 10.1002/dvg.20205

[LIAOGAD298703C6] Botchkareva NV, Khlgatian M, Longley BJ, Botchkarev VA, Gilchrest BA. 2001 SCF/c-kit signaling is required for cyclic regeneration of the hair pigmentation unit. FASEB J 15: 645–658. 11259383 10.1096/fj.00-0368com

[LIAOGAD298703C7] Brannan CI, Lyman SD, Williams DE, Eisenman J, Anderson DM, Cosman D, Bedell MA, Jenkins NA, Copeland NG. 1991 Steel-Dickie mutation encodes a c-kit ligand lacking transmembrane and cytoplasmic domains. Proc Natl Acad Sci 88: 4671–4674. 1711207 10.1073/pnas.88.11.4671 PMC51727

[LIAOGAD298703C8] Chang CY, Pasolli HA, Giannopoulou EG, Guasch G, Gronostajski RM, Elemento O, Fuchs E. 2013 NFIB is a governor of epithelial–melanocyte stem cell behaviour in a shared niche. Nature 495: 98–102. 23389444 10.1038/nature11847 PMC3635831

[LIAOGAD298703C9] Chen Z, Liu C, Patel AJ, Liao CP, Wang Y, Le LQ. 2014 Cells of origin in the embryonic nerve roots for NF1-associated plexiform neurofibroma. Cancer Cell 26: 695–706. 25446898 10.1016/j.ccell.2014.09.009 PMC4254535

[LIAOGAD298703C10] Copeland NG, Gilbert DJ, Cho BC, Donovan PJ, Jenkins NA, Cosman D, Anderson D, Lyman SD, Williams DE. 1990 Mast cell growth factor maps near the steel locus on mouse chromosome 10 and is deleted in a number of steel alleles. Cell 63: 175–183. 1698554 10.1016/0092-8674(90)90298-s

[LIAOGAD298703C11] Cotsarelis G, Sun TT, Lavker RM. 1990 Label-retaining cells reside in the bulge area of pilosebaceous unit: implications for follicular stem cells, hair cycle, and skin carcinogenesis. Cell 61: 1329–1337. 2364430 10.1016/0092-8674(90)90696-c

[LIAOGAD298703C12] Coulombe PA, Kopan R, Fuchs E. 1989 Expression of keratin K14 in the epidermis and hair follicle: insights into complex programs of differentiation. J Cell Biol 109: 2295–2312. 2478566 10.1083/jcb.109.5.2295 PMC2115845

[LIAOGAD298703C13] Dassule HR, Lewis P, Bei M, Maas R, McMahon AP. 2000 Sonic hedgehog regulates growth and morphogenesis of the tooth. Development 127: 4775–4785. 11044393 10.1242/dev.127.22.4775

[LIAOGAD298703C14] Decker L, Desmarquet-Trin-Dinh C, Taillebourg E, Ghislain J, Vallat JM, Charnay P. 2006 Peripheral myelin maintenance is a dynamic process requiring constant Krox20 expression. J Neurosci 26: 9771–9779. 16988048 10.1523/JNEUROSCI.0716-06.2006 PMC6674452

[LIAOGAD298703C15] Ding L, Saunders TL, Enikolopov G, Morrison SJ. 2012 Endothelial and perivascular cells maintain haematopoietic stem cells. Nature 481: 457–462. 22281595 10.1038/nature10783 PMC3270376

[LIAOGAD298703C16] Driskell RR, Clavel C, Rendl M, Watt FM. 2011 Hair follicle dermal papilla cells at a glance. J Cell Sci 124: 1179–1182. 21444748 10.1242/jcs.082446 PMC3115771

[LIAOGAD298703C17] Gambardella L, Schneider-Maunoury S, Voiculescu O, Charnay P, Barrandon Y. 2000 Pattern of expression of the transcription factor Krox-20 in mouse hair follicle. Mech Dev 96: 215–218. 10960786 10.1016/s0925-4773(00)00398-1

[LIAOGAD298703C18] Gokbuget D, Pereira JA, Bachofner S, Marchais A, Ciaudo C, Stoffel M, Schulte JH, Suter U. 2015 The Lin28/let-7 axis is critical for myelination in the peripheral nervous system. Nat Commun 6: 8584. 26466203 10.1038/ncomms9584 PMC4634210

[LIAOGAD298703C19] Hayashi S, McMahon AP. 2002 Efficient recombination in diverse tissues by a tamoxifen-inducible form of Cre: a tool for temporally regulated gene activation/inactivation in the mouse. Dev Biol 244: 305–318. 11944939 10.1006/dbio.2002.0597

[LIAOGAD298703C20] Hsu YC, Pasolli HA, Fuchs E. 2011 Dynamics between stem cells, niche, and progeny in the hair follicle. Cell 144: 92–105. 21215372 10.1016/j.cell.2010.11.049 PMC3050564

[LIAOGAD298703C21] Hsu YC, Li L, Fuchs E. 2014 Transit-amplifying cells orchestrate stem cell activity and tissue regeneration. Cell 157: 935–949. 24813615 10.1016/j.cell.2014.02.057 PMC4041217

[LIAOGAD298703C22] Huelsken J, Vogel R, Erdmann B, Cotsarelis G, Birchmeier W. 2001 β-Catenin controls hair follicle morphogenesis and stem cell differentiation in the skin. Cell 105: 533–545. 11371349 10.1016/s0092-8674(01)00336-1

[LIAOGAD298703C23] Kunisada T, Lu SZ, Yoshida H, Nishikawa S, Nishikawa S, Mizoguchi M, Hayashi S, Tyrrell L, Williams DA, Wang X, 1998a Murine cutaneous mastocytosis and epidermal melanocytosis induced by keratinocyte expression of transgenic stem cell factor. J Exp Med 187: 1565–1573. 9584135 10.1084/jem.187.10.1565 PMC2212288

[LIAOGAD298703C24] Kunisada T, Yoshida H, Yamazaki H, Miyamoto A, Hemmi H, Nishimura E, Shultz LD, Nishikawa S, Hayashi S. 1998b Transgene expression of steel factor in the basal layer of epidermis promotes survival, proliferation, differentiation and migration of melanocyte precursors. Development 125: 2915–2923. 9655813 10.1242/dev.125.15.2915

[LIAOGAD298703C25] Le LQ, Shipman T, Burns DK, Parada LF. 2009 Cell of origin and microenvironment contribution for NF1-associated dermal neurofibromas. Cell Stem Cell 4: 453–463. 19427294 10.1016/j.stem.2009.03.017 PMC2737469

[LIAOGAD298703C26] Lee P, Morley G, Huang Q, Fischer A, Seiler S, Horner JW, Factor S, Vaidya D, Jalife J, Fishman GI. 1998 Conditional lineage ablation to model human diseases. Proc Natl Acad Sci 95: 11371–11376. 9736743 10.1073/pnas.95.19.11371 PMC21649

[LIAOGAD298703C27] Lennartsson J, Ronnstrand L. 2012 Stem cell factor receptor/c-Kit: from basic science to clinical implications. Physiol Rev 92: 1619–1649. 23073628 10.1152/physrev.00046.2011

[LIAOGAD298703C28] Leone DP, Genoud S, Atanasoski S, Grausenburger R, Berger P, Metzger D, Macklin WB, Chambon P, Suter U. 2003 Tamoxifen-inducible glia-specific Cre mice for somatic mutagenesis in oligodendrocytes and Schwann cells. Mol Cell Neurosci 22: 430–440. 12727441 10.1016/s1044-7431(03)00029-0

[LIAOGAD298703C29] Li S, Miao T, Sebastian M, Bhullar P, Ghaffari E, Liu M, Symonds AL, Wang P. 2012 The transcription factors Egr2 and Egr3 are essential for the control of inflammation and antigen-induced proliferation of B and T cells. Immunity 37: 685–696. 23021953 10.1016/j.immuni.2012.08.001 PMC3477314

[LIAOGAD298703C30] Lin JY, Fisher DE. 2007 Melanocyte biology and skin pigmentation. Nature 445: 843–850. 17314970 10.1038/nature05660

[LIAOGAD298703C31] McGill GG, Horstmann M, Widlund HR, Du J, Motyckova G, Nishimura EK, Lin YL, Ramaswamy S, Avery W, Ding HF, 2002 Bcl2 regulation by the melanocyte master regulator Mitf modulates lineage survival and melanoma cell viability. Cell 109: 707–718. 12086670 10.1016/s0092-8674(02)00762-6

[LIAOGAD298703C32] Nishimura EK, Jordan SA, Oshima H, Yoshida H, Osawa M, Moriyama M, Jackson IJ, Barrandon Y, Miyachi Y, Nishikawa S. 2002 Dominant role of the niche in melanocyte stem-cell fate determination. Nature 416: 854–860. 11976685 10.1038/416854a

[LIAOGAD298703C33] Nishimura EK, Granter SR, Fisher DE. 2005 Mechanisms of hair graying: incomplete melanocyte stem cell maintenance in the niche. Science 307: 720–724. 15618488 10.1126/science.1099593

[LIAOGAD298703C34] Nishimura EK, Suzuki M, Igras V, Du J, Lonning S, Miyachi Y, Roes J, Beermann F, Fisher DE. 2010 Key roles for transforming growth factor β in melanocyte stem cell maintenance. Cell Stem Cell 6: 130–140. 20144786 10.1016/j.stem.2009.12.010 PMC3437996

[LIAOGAD298703C35] Okura M, Maeda H, Nishikawa S, Mizoguchi M. 1995 Effects of monoclonal anti-c-kit antibody (ACK2) on melanocytes in newborn mice. J Invest Dermatol 105: 322–328. 7545201 10.1111/1523-1747.ep12319939

[LIAOGAD298703C36] Peters EM, Tobin DJ, Botchkareva N, Maurer M, Paus R. 2002 Migration of melanoblasts into the developing murine hair follicle is accompanied by transient c-Kit expression. J Histochem Cytochem 50: 751–766. 12019292 10.1177/002215540205000602

[LIAOGAD298703C37] Plikus MV, Chuong CM. 2008 Complex hair cycle domain patterns and regenerative hair waves in living rodents. J Invest Dermatol 128: 1071–1080. 18094733 10.1038/sj.jid.5701180 PMC2705329

[LIAOGAD298703C38] Plikus MV, Chuong CM. 2014 Macroenvironmental regulation of hair cycling and collective regenerative behavior. Cold Spring Harb Perspect Med 4: a015198. 24384813 10.1101/cshperspect.a015198 PMC3869280

[LIAOGAD298703C39] Plikus MV, Gay DL, Treffeisen E, Wang A, Supapannachart RJ, Cotsarelis G. 2012 Epithelial stem cells and implications for wound repair. Semin Cell Dev Biol 23: 946–953. 23085626 10.1016/j.semcdb.2012.10.001 PMC3518754

[LIAOGAD298703C40] Plikus MV, Mayer JA, de la Cruz D, Baker RE, Maini PK, Maxson R, Chuong CM. 2008 Cyclic dermal BMP signalling regulates stem cell activation during hair regeneration. Nature 451: 340–344. 18202659 10.1038/nature06457 PMC2696201

[LIAOGAD298703C41] Rabbani P, Takeo M, Chou W, Myung P, Bosenberg M, Chin L, Taketo MM, Ito M. 2011 Coordinated activation of Wnt in epithelial and melanocyte stem cells initiates pigmented hair regeneration. Cell 145: 941–955. 21663796 10.1016/j.cell.2011.05.004 PMC3962257

[LIAOGAD298703C42] Reith AD, Rottapel R, Giddens E, Brady C, Forrester L, Bernstein A. 1990 W mutant mice with mild or severe developmental defects contain distinct point mutations in the kinase domain of the c-kit receptor. Genes Dev 4: 390–400. 1692559 10.1101/gad.4.3.390

[LIAOGAD298703C43] Saint-Jeannet JP, He X, Varmus HE, Dawid IB. 1997 Regulation of dorsal fate in the neuraxis by Wnt-1 and Wnt-3a. Proc Natl Acad Sci 94: 13713–13718. 9391091 10.1073/pnas.94.25.13713 PMC28371

[LIAOGAD298703C44] Samuelov L, Sprecher E, Tsuruta D, Biro T, Kloepper JE, Paus R. 2012 P-cadherin regulates human hair growth and cycling via canonical Wnt signaling and transforming growth factor-β2. J Invest Dermatol 132: 2332–2341. 22696062 10.1038/jid.2012.171

[LIAOGAD298703C45] Schneider-Maunoury S, Topilko P, Seitandou T, Levi G, Cohen-Tannoudji M, Pournin S, Babinet C, Charnay P. 1993 Disruption of Krox-20 results in alteration of rhombomeres 3 and 5 in the developing hindbrain. Cell 75: 1199–1214. 7903221 10.1016/0092-8674(93)90329-o

[LIAOGAD298703C46] Schouwey K, Delmas V, Larue L, Zimber-Strobl U, Strobl LJ, Radtke F, Beermann F. 2007 Notch1 and Notch2 receptors influence progressive hair graying in a dose-dependent manner. Dev Dyn 236: 282–289. 17080428 10.1002/dvdy.21000

[LIAOGAD298703C47] Sharov AA, Sharova TY, Mardaryev AN, Tommasi di Vignano A, Atoyan R, Weiner L, Yang S, Brissette JL, Dotto GP, Botchkarev VA. 2006 Bone morphogenetic protein signaling regulates the size of hair follicles and modulates the expression of cell cycle-associated genes. Proc Natl Acad Sci 103: 18166–18171. 17114283 10.1073/pnas.0608899103 PMC1838724

[LIAOGAD298703C48] Shimomura Y, Christiano AM. 2010 Biology and genetics of hair. Annu Rev Genomics Hum Genet 11: 109–132. 20590427 10.1146/annurev-genom-021610-131501

[LIAOGAD298703C49] Shyh-Chang N, Daley GQ. 2013 Lin28: primal regulator of growth and metabolism in stem cells. Cell Stem Cell 12: 395–406. 23561442 10.1016/j.stem.2013.03.005 PMC3652335

[LIAOGAD298703C50] Shyh-Chang N, Zhu H, Yvanka de Soysa T, Shinoda G, Seligson MT, Tsanov KM, Nguyen L, Asara JM, Cantley LC, Daley GQ. 2013 Lin28 enhances tissue repair by reprogramming cellular metabolism. Cell 155: 778–792. 24209617 10.1016/j.cell.2013.09.059 PMC3917449

[LIAOGAD298703C51] Skucas VA, Duffy AM, Harte-Hargrove LC, Magagna-Poveda A, Radman T, Chakraborty G, Schroeder CE, MacLusky NJ, Scharfman HE. 2013 Testosterone depletion in adult male rats increases mossy fiber transmission, LTP, and sprouting in area CA3 of hippocampus. J Neurosci 33: 2338–2355. 23392664 10.1523/JNEUROSCI.3857-12.2013 PMC3711621

[LIAOGAD298703C52] Solanas G, Benitah SA. 2013 Regenerating the skin: a task for the heterogeneous stem cell pool and surrounding niche. Nat Rev Mol Cell Biol 14: 737–748. 24064540 10.1038/nrm3675

[LIAOGAD298703C53] Topilko P, Schneider-Maunoury S, Levi G, Baron-Van Evercooren A, Chennoufi AB, Seitanidou T, Babinet C, Charnay P. 1994 Krox-20 controls myelination in the peripheral nervous system. Nature 371: 796–799. 7935840 10.1038/371796a0

[LIAOGAD298703C54] Tumbar T, Guasch G, Greco V, Blanpain C, Lowry WE, Rendl M, Fuchs E. 2004 Defining the epithelial stem cell niche in skin. Science 303: 359–363. 14671312 10.1126/science.1092436 PMC2405920

[LIAOGAD298703C55] Vasioukhin V, Degenstein L, Wise B, Fuchs E. 1999 The magical touch: genome targeting in epidermal stem cells induced by tamoxifen application to mouse skin. Proc Natl Acad Sci 96: 8551–8556. 10411913 10.1073/pnas.96.15.8551 PMC17554

[LIAOGAD298703C56] Veis DJ, Sorenson CM, Shutter JR, Korsmeyer SJ. 1993 Bcl-2-deficient mice demonstrate fulminant lymphoid apoptosis, polycystic kidneys, and hypopigmented hair. Cell 75: 229–240. 8402909 10.1016/0092-8674(93)80065-m

[LIAOGAD298703C57] Virador V, Matsunaga N, Matsunaga J, Valencia J, Oldham RJ, Kameyama K, Peck GL, Ferrans VJ, Vieira WD, Abdel-Malek ZA, 2001 Production of melanocyte-specific antibodies to human melanosomal proteins: expression patterns in normal human skin and in cutaneous pigmented lesions. Pigment Cell Res 14: 289–297. 11549113 10.1034/j.1600-0749.2001.140410.x

[LIAOGAD298703C58] Voiculescu O, Charnay P, Schneider-Maunoury S. 2000 Expression pattern of a Krox-20/Cre knock-in allele in the developing hindbrain, bones, and peripheral nervous system. Genesis 26: 123–126. 10686605 10.1002/(sici)1526-968x(200002)26:2<123::aid-gene7>3.0.co;2-o

[LIAOGAD298703C59] Watt FM. 2014 Mammalian skin cell biology: at the interface between laboratory and clinic. Science 346: 937–940. 25414300 10.1126/science.1253734

[LIAOGAD298703C60] Yoshida H, Kunisada T, Grimm T, Nishimura EK, Nishioka E, Nishikawa SI. 2001 Review: melanocyte migration and survival controlled by SCF/c-kit expression. J Investig Dermatol Symp Proc 6: 1–5. 10.1046/j.0022-202x.2001.00006.x 11764276

[LIAOGAD298703C61] Zorick TS, Syroid DE, Brown A, Gridley T, Lemke G. 1999 Krox-20 controls SCIP expression, cell cycle exit and susceptibility to apoptosis in developing myelinating Schwann cells. Development 126: 1397–1406. 10068633 10.1242/dev.126.7.1397

