# Supplementary material for: Identification of hair shaft progenitors that create a niche for hair pigmentation
Source: Genes Dev. 2017 Apr 15;31(8):744–56. doi: 10.1101/gad.298703.117 (PMC5435888; doi:10.1101/gad.298703.117)
Supplement: Supplemental Material [file supp_31_8_744__index.html]

Identification of hair shaft progenitors that create a niche for hair pigmentation — Supplemental Material 

# Identification of hair shaft progenitors that create a niche for hair pigmentation

## Supplemental Material

- Supplemental\_Figures\_and\_Legends.pdf
