# Supplementary material for: Identification of hair shaft progenitors that create a niche for hair pigmentation
Source: Genes Dev. 2017 Apr 15;31(8):744–56. doi: 10.1101/gad.298703.117 (PMC5435888; doi:10.1101/gad.298703.117)

Supplemental Figure Legends

Identification of Hair Shaft Progenitors That Create A Niche for Hair Pigmentation

Chung-Ping Liao [1], Reid C. Booker [1], Sean J. Morrison [2,3,4,5], and Lu Q. Le [1,3,4]

[1]Department of Dermatology, [2]Department of Pediatrics and Children's Research Institute, [3]Simmons Comprehensive Cancer Center, [4]Hamon Center for Regenerative Science and Medicine. University of Texas Southwestern Medical Center, Dallas, TX 75390-9133, USA. [5]Howard Hughes Medical Institute

Figure S1. *Scf$^{flox/gfp}$; Krox20Cre* Mice Displayed Progressive Hair Graying.

(A) The coat color change along aging in a representative *Scf$^{flox/gfp}$; Krox20Cre* mouse (top) as compared to a littermate *Scf$^{flox/gfp}$* control (bottom) (n = 20).

(B) The coat color and hair mount image from a P90 *Scf$^{flox/gfp}$; Krox20Cre* mouse. Note the depigmented hairs in the proximal end and partially pigmented hair in the distal end. Circle indicated the location where hairs were plucked from. Arrow head indicted the depigmented hairs.

(C) The coat color and hair mount image from a P145 *Scf$^{flox/gfp}$; Krox20Cre* mouse. Note all the hairs were almost entirely depigmented. Circle indicated the location where hairs were plucked from. Arrow head indicted the depigmented hairs.

Figure S2. Induction of SCF Depletion in Schwann Cells or Melanocytes Does Not Affect Hair Pigmentation.

(A) Depletion of SCF in *PLP*-lineage Schwann cell does not affect hair pigmentation. *Scf*$^{flox/gfp}$*; PLPCre*^{ERT2} mice with 4-hydroxytamoxifen induction at P0 exhibited normal hair pigmentation (n = 4).

(B) Whole mount LacZ staining revealed that *PLP*-lineage cells (arrow head) were present in subcutaneous tissue, demonstrating the specificity and active Cre mediated recombination.

(C) Tissue section of (B) revealed that *PLP*-lineage cells (arrow head) were present in subcutaneous nerves.

(D) Induction of SCF depletion in Schwann cells in adult mice does not affect hair pigmentation. *Scf*$^{flox/gfp}$; *PLPCre*^{ERT2} mice were depilated followed by tamoxifen treatment, no hair color change was noticed.

(E) Induction of SCF depletion in melanocytes in adult mice does not affect hair pigmentation. *Scf*$^{flox/gfp}$; *TyrCre*^{ERT2} mice were depilated followed by tamoxifen treatment, no hair color change was noticed.

(F) Induction of SCF depletion systemically in adult mice causes hair hypopigmentation. *Scf*$^{flox/flox}$; *CMVCre*^{ERT} mice were depilated followed by tamoxifen treatment, newly regenerated hairs drastically lost pigmentation.

(G) To ascertain the hypopigmented hairs in (F) are a result a tamoxifen treatment, vehicle control was applied. Only a small portion of hypopigmented hairs was observed, this is likely due to the minor leak of strong *CMV* promoter.

Scale bar, 200 µm.

Figure S3. Induction of *Scf* Gene Ablation in HF Stem Cells Causes Hair Hypopigmentation.

(A) Induction of SCF ablation in HF stem cells during anagen results in a late-onset hair hypopigmentation. At P0 new born stage (HF growing at anagen), $Scf^{flox/gfp}$; $K14Cre^{ERT}$ mice were treated with 4-hydroxytamoxifen to induce SCF ablation in *K14*-lineage cells. These mice develop normally pigmented coat initially; however, their hairs turned gray after second hair cycle (n = 5).

(B) Induction of SCF ablation in HF stem cells during telogen results in an immediate hair hypopigmentation. At 3-month-old adult stage (HF resting at telogen), $Scf^{flox/gfp}$; $K14Cre^{ERT}$ mice were depilated followed by tamoxifen treatment for 5 consecutive days to induce SCF ablation in *K14*-lineage cells. These mice developed hypopigmented hairs immediately during hair regeneration (n = 4).

(C) To ascertain the hypopigmented hairs in (B) is not caused by possibly leaky $K14Cre^{ERT}$, $Scf^{flox/gfp}$; $K14Cre^{ERT}$ mice were treated by vehicle and no coat color change was noticed (n = 2).

(D) To ascertain the hypopigmented hairs in (B) is not caused by tamoxifen treatment, $Scf^{flox/gfp}$ mice were treated by tamoxifen and no coat color change was noticed (n = 2).

(E) Illustration of hair color change when induction of SCF depletion occurs at different hair developmental stages.

Liao_298703_Fig.S1

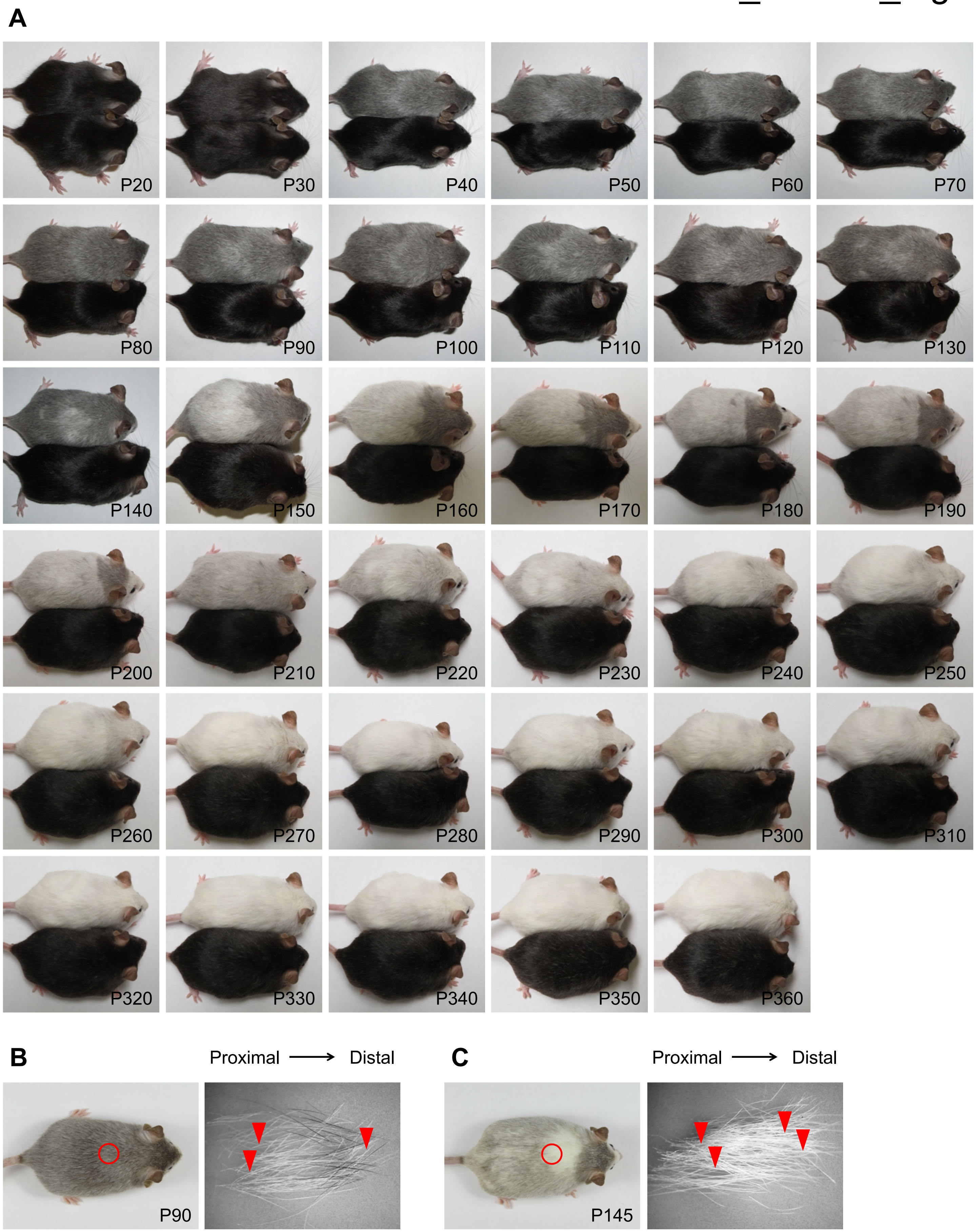

Liao_298703_Fig.S2

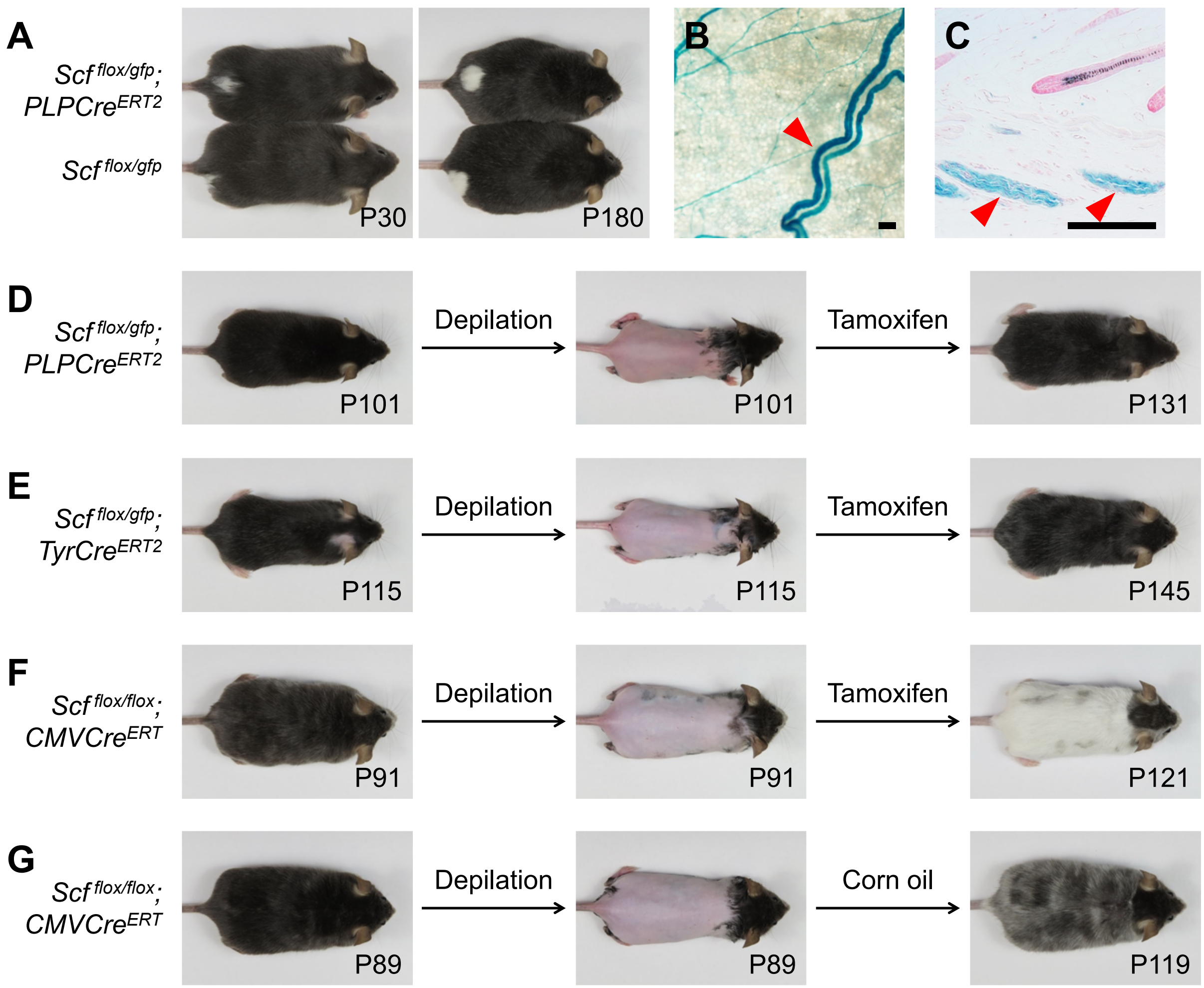

Liao_298703_Fig.S3

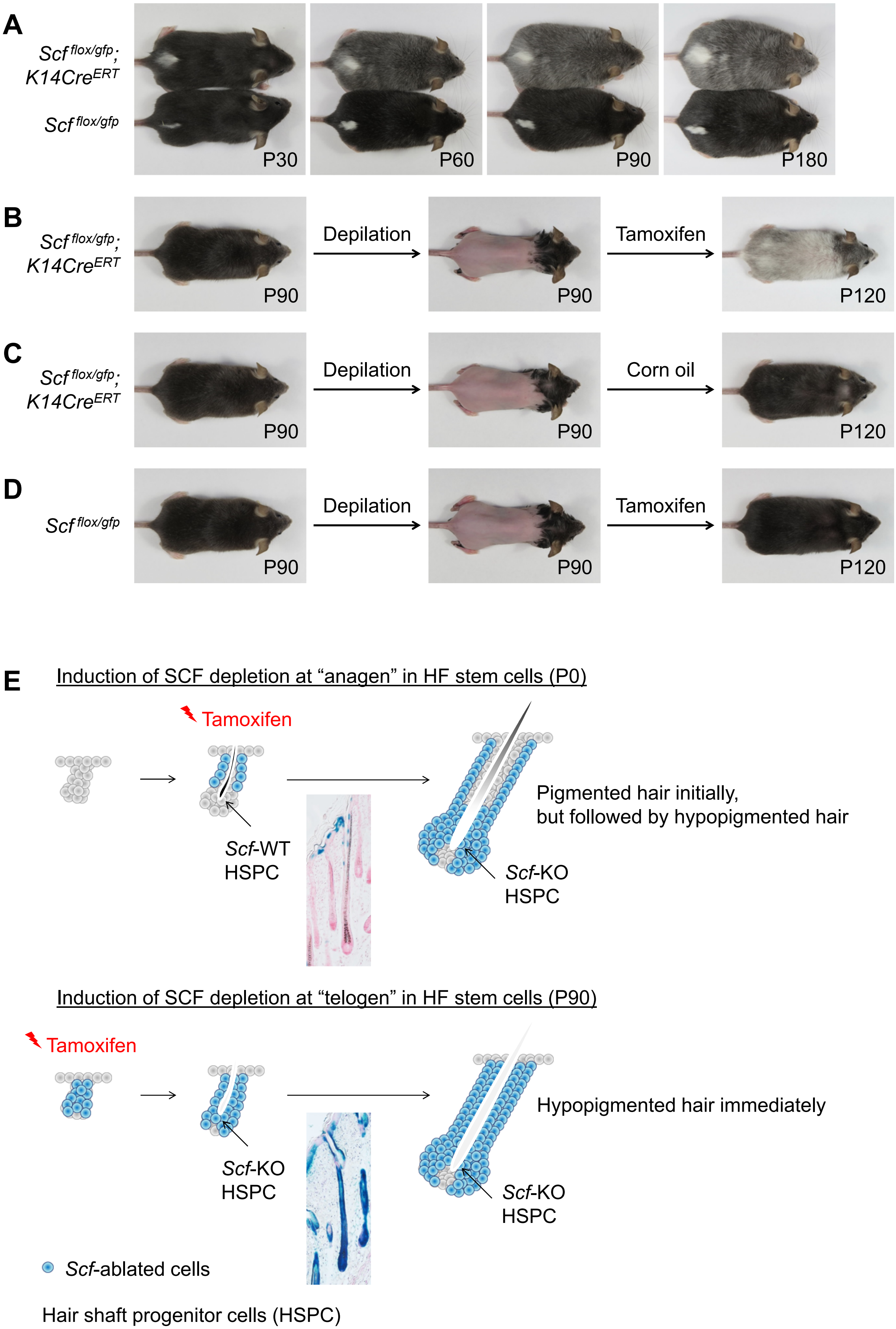

Supplement: Supplemental Material [file supp_gad.298703.117_Supplemental_Figures_and_Legends.pdf]
